# A Complex Case of Emphysematous Cystitis in a Peritoneal Dialysis Patient

**DOI:** 10.1155/2021/8343022

**Published:** 2021-07-07

**Authors:** Christina Okello, Rajesh Raj

**Affiliations:** ^1^Launceston General Hospital, 274-280 Charles Street, Launceston 7250, Tasmania, Australia; ^2^Department of Nephrology, Launceston General Hospital, 274-280 Charles Street, Launceston 7250, Tasmania, Australia

## Abstract

Emphysematous cystitis (EC) is a relatively rare condition characterized by gas formation in the bladder wall and/or lumen. We report a case of emphysematous cystitis with a bladder perforation in an 84-year-old male on peritoneal dialysis who presented with fever, dysuria, hematuria, and hypotension. Gas in the bladder wall, as well as a small perforation in the roof of the urinary bladder, was seen on the abdominal CT scan. The causative organism identified was *Escherichia coli.* The patient recovered with broad-spectrum antibiotics along with bladder irrigation and drainage. After initial bladder washouts, peritoneal dialysis was continued with close monitoring. Early antibiotic therapy and a conservative approach to the management of small intraperitoneal bladder perforations were effective in this patient. Peritoneal dialysis was uninterrupted for the duration of the admission and after discharge.

## 1. Introduction

Of all gas-forming infections of the urinary tract, emphysematous cystitis (EC) is the most common [1]. Patients with diabetic cystopathy, neurogenic bladder, and urinary stasis secondary to urethral strictures or bladder outlet obstruction are more susceptible. Various gas-forming bacterial and fungal organisms have been implicated in the pathogenesis, with *Escherichia coli* (*E. coli*) being the most common culprit [2]. Computerized tomographic (CT) scans are the gold standard in the diagnosis of this condition [3]. Rupture of the bladder is an uncommon complication of EC that may have grave consequences if not diagnosed and treated early [4]. For smaller intraperitoneal bladder ruptures, recent reports have shown that a conservative approach may be sufficient if urinary antibiotic prophylaxis is continued after initial antibiotic therapy, and continued urinary drainage through an indwelling catheter is provided for a minimum of two weeks, until closure of the perforation is evident on the CT cystogram [5–8]. Similarly, certain international guidelines have recently adopted a conservative approach in the management of small, uncomplicated intraperitoneal bladder perforations [9].

## 2. Case Report

An 84-year-old male patient was admitted to our hospital with a fever, increased frequency of micturition, and hematuria for 2 days. He reported dysuria and cloudy, foul-smelling urine. He had no abdominal pain but was noted to be hypotensive at presentation. He had recently been discharged from hospital on anticoagulation for an unprovoked pulmonary embolism two weeks prior. He had end-stage kidney disease secondary to biopsy-confirmed mesangial-proliferative glomerulonephritis for which he had been on intermittent peritoneal dialysis (P.D) for 5 years. His other comorbidities included chronic retention due to benign prostate hypertrophy for which he was on tamsulosin/dutasteride, recurrent urinary tract infections (UTIs), a prior history of urolithiasis, hypertension, rheumatoid arthritis, hypercholesterolemia, and osteoarthritis of the spine.

On examination, he was febrile (38°C) and hypotensive at 66/42 mmHg. A clean peritoneal dialysis catheter site draining clear effluent was noted on inspection. There was no guarding on palpation of the abdomen, but there was mild suprapubic tenderness.

Resuscitation with intravenous fluids improved blood pressure to 132/78 mmHg.

Laboratory results, as shown in Table 1, revealed an elevated C-reactive protein (CRP) and thrombocytopenia. His Hba1c was 5.8%.

**Figure 1:**
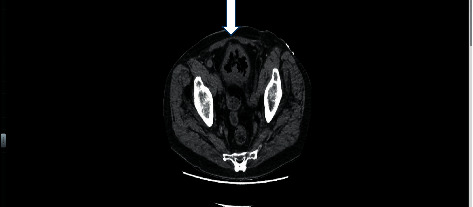
CT scan image showing a small perforation at the bladder dome and changes in keeping with emphysematous cystitis.

**Figure 2:**
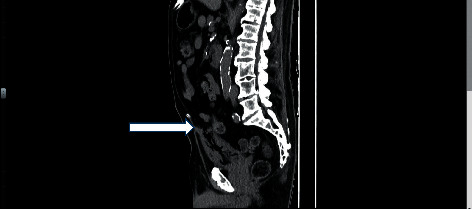
Axial view of the CT scan image demonstrating small bladder perforation and emphysematous cystitis.

Urinalysis revealed macroscopic hematuria, pyuria, and bacteriuria. A noncontrast abdominal Computed Tomography (CT) scan was performed (Figures 1 and 2). It revealed prostatomegaly with marked intraluminal and small-volume intramural gas within a thick-walled urinary bladder. There was also a small localized perforation within the dome of the urinary bladder. Findings suggested bladder outlet obstruction and emphysematous cystitis.

The patient's anticoagulant medication was ceased after discussing with the patient the risks of ongoing bleeding and clot recurrence. An indwelling catheter was inserted, and continuous bladder irrigation commenced. Intravenous piperacillin-tazobactam was started after blood and urine cultures were taken. Peritoneal fluid showed no discoloration or blood. Dialysis was continued with regular monitoring of fluid appearance. He was also commenced on antifungal prophylaxis against P.D peritonitis.

*Escherichia coli* was grown in the urine culture. Blood cultures were persistently negative.

The patient's clinical status progressively improved. He was afebrile by day 3 and discharged ten days later. Peritoneal dialysis was uninterrupted for the duration of the admission and after discharge.

**Figure 3:**
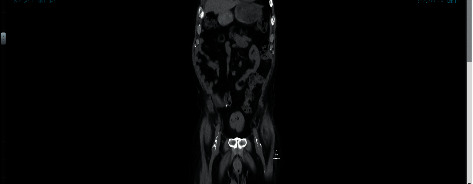
CT scan image demonstrating emphysematous cystitis with no demonstration of bladder perforation.

**Figure 4:**
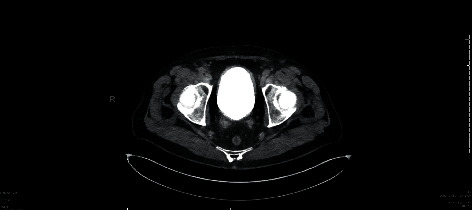
CT cystogram image displaying a contrast filled bladder. No extravasation/leakage of contrast from the bladder was noted.

Although a repeat CT scan on day 7 revealed persistent intraluminal and intramural gas (Figure 3), these findings had completely resolved 2 weeks after discharge (Figure 4).

## 3. Discussion

Our report highlights a potentially fatal infection that presented unexpectedly in a gentleman on peritoneal dialysis. Despite the appearance of a bladder perforation on the CT scan, there were no signs of peritonitis, and the patient's urine could be constantly drained allowing us to safely continue dialysis treatment.

The majority of reported patients affected by EC are female, elderly, and diabetic. The clinical presentation can range from asymptomatic to severely symptomatic, including presentations such as peritonitis or septic shock [2]. In addition to the typical symptoms of cystitis, some patients report the unique presence of pneumaturia [10].

The exact mechanism of gas formation in emphysematous infections is unclear. It is presumed to be due to the presence of gas-forming organisms in a diabetic milieu that rapidly ferment glucose to produce carbon dioxide. In nondiabetic patients, it has been proposed that urinary lactose or tissue proteins may serve as substrates for the gas formation. Patients with recurrent urinary tract infections, indwelling urethral catheters, neurogenic bladders, and immunosuppressive conditions are predisposed to complicated urinary tract infections, including EC [1].

Urinary bladder wall perforation as a result of EC is extremely rare. There have been case reports of EC due to non-*Albicans candida* species that required surgery [11, 12] as well as reports of *E. coli* EC managed conservatively [4, 13, 14]. Our patient had a small bladder perforation in the dome of the bladder and mild prostatomegaly on his CT scan. The presence of obstructive voiding worsened by high intravesical pressure in a distended bladder could account for the rupture of the “fragile” and inflamed bladder wall [5].

Conservative management protects patients from anaesthetic risks and surgical complications [6]. Nonoperative management of bladder perforation could either be in the form of insertion of an indwelling transurethral Foley catheter alone or a combination of bladder and peritoneal drainage [8]. Studies have shown that nonoperative treatment for intraperitoneal bladder rupture is appropriate if there is no associated organ injury that requires laparotomy and if there is availability of adequate bladder drainage and no complications such as peritonitis [4, 14], all features that were achieved in this patient.

## 4. Conclusions

This case demonstrates the conservative management of a patient on peritoneal dialysis who developed emphysematous cystitis and bladder perforation as a complication of *E. coli* urinary infection in the setting of bladder outlet obstruction.

Peritoneal dialysis was largely uninterrupted for the duration of the infection and even after resolution of the same.

The prognosis of EC is favorable with prompt diagnosis and timely commencement of treatment. Multidisciplinary care is key in managing complex cases of emphysematous cystitis.

## Figures and Tables

**Table 1:** Laboratory test results from the first week of admission.

	Day 1	Day 2	Day 3	Day 4	Day 5	Day 6	Day 7
Haemoglobin (g/dl)	147	148	151	146	146	147	151
White cell count (^∗^10^9^)	7.6	6.0	6.4	6.1	5.3	6.0	5.2
Platelets (^∗^10^9^)	84	90	111	106	105	114	119
C-reactive protein	55	88	58	41	35	26	

## Data Availability

The data used during the current case report are available from the corresponding author upon reasonable request.
